# Supplementary material for: Brown trout (Salmo trutta) originating from warmer streams in Iceland exhibit increased energetic efficiency
Source: Commun Biol. 2026 Mar 31;9:710. doi: 10.1038/s42003-026-09911-5 (PMC13201538; doi:10.1038/s42003-026-09911-5)
Supplement: Supplementary file 2 — Supplementary Information [file 42003_2026_9911_MOESM2_ESM.pdf]

Supplementary Information: Brown trout (*Salmo trutta*) originating from warmer streams in Iceland exhibit increased energetic efficiency

Eoin J. O'Gorman[1,†,*], Alexia M. González-Ferreras[2,1,†], Penelope S.A. Blyth[3,4], Jamie Coughlan[5], Jack Hawksley[3], Phil McGinnity[5], Karl P. Phillips[5,6], Thomas E. Reed[5,*]

[1] *School of Life Sciences, University of Essex, Wivenhoe Park, Colchester, CO4 3SQ, UK*

[2] *IHCantabria - Instituto de Hidráulica Ambiental de la Universidad de Cantabria. C/Isabel Torres 15, 39011, Santander, Spain*

[3] *Department of Life Sciences, Imperial College London, Silwood Park Campus, Buckhurst Road, Ascot, Berkshire SL5 7PY, UK*

[4] *School of Biosciences, University of Sheffield, Sheffield, S10 2TN, UK*

[5] *School of Biological, Earth and Environmental Sciences, University College Cork, Distillery Fields, North Mall, Cork, Ireland*

[6] *Canadian Rivers Institute, University of New Brunswick, Fredericton, NB E3B 5A3, Canada*

[†] *These authors contributed equally*

[*] *Correspondence to e.ogorman@essex.ac.uk and treed@ucc.ie*

List of ORCIDs

EJOG: 0000-0003-4507-5690

AMGF: 0000-0002-1039-6737

PSAB: 0000-0003-3071-9668

KPP: 0000-0002-8557-0293

TER: 0000-0002-2993-0477

Supplementary Methods

Within-stream genetic diversity

Basic population genetic summary statistics (number of alleles, allelic richness, observed (H_o) and expected (H_e) heterozygosity) were calculated for each of the three streams using the '*divBasic*' function in the '*diveRsity*' package in R, on a per-marker as well as overall (across all markers) basis. This function also returns Fisher's exact tests for conformance with Hardy–Weinberg equilibrium (HWE) for each marker (following 10,000 Monte Carlo replicates), which were used for post-hoc marker quality control within streams.

Supplementary Results

Within-stream genetic diversity

Genetic diversity was low within all three streams, with the number of alleles ranging from 1-7, and an average allelic richness of 3.21 (Table S9). Observed heterozygosity was similar in each stream, with an overall average H_o of 0.54, 0.54 and 0.53 in IS12, IS1, and IS5, respectively. Some loci (markers One102-a, ppStr3, Ssa197, and SaSaTAP2A) exhibited considerably lower heterozygosity (Table S9). Only three of the 51 Fisher's exact tests (17 markers × 3 streams) showed a significant departure from HWE (Table S9).

Supplementary Figure 1. Effects of source-stream temperature on metabolic rate considering three separate populations of trout. The optimum model describing variation in the metabolic rate of brown trout included main effects of (a) temperature and (b) body mass, but no significant main or interactive effects of source-stream temperature (Linear regression: $y = -1.098 + 0.7986 \times \log(M) + 0.3693 \times T - T_0/kTT_0$; $F_{2,83} = 86.49$; $p < 0.001$; $r^2 = 0.67$). The graphical legend indicates the stream of origin for each population: blue = IS12, orange = IS1, and red = IS5. Shaded areas are the 95% confidence intervals around the fitted regression lines.

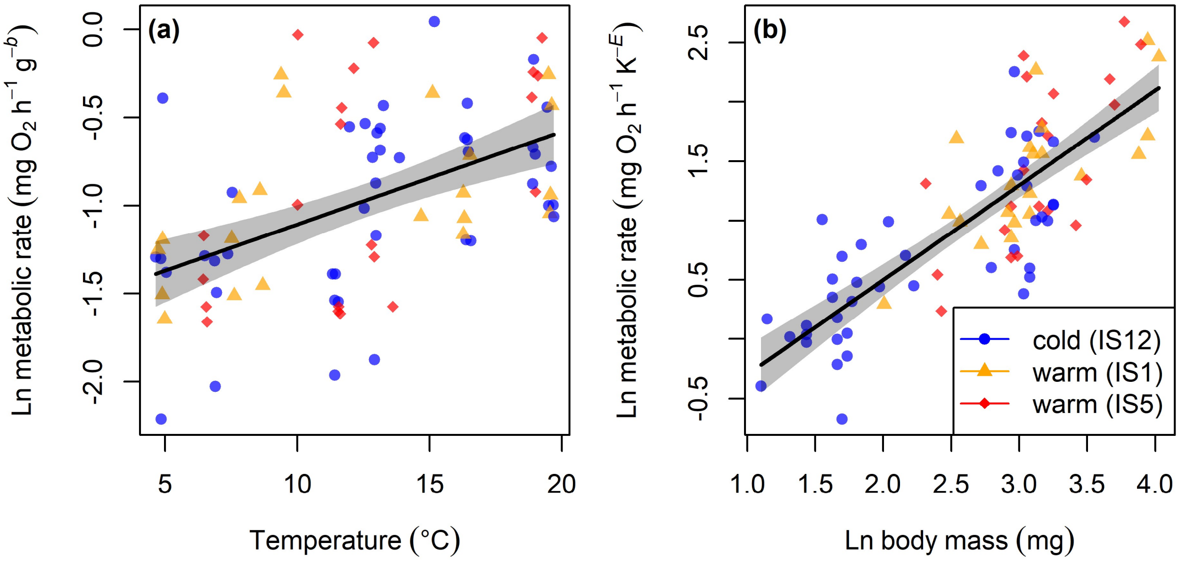

Supplementary Figure 2. Effects of source-stream temperature on feeding rate considering three separate populations of trout. The optimum model describing variation in the feeding rate of brown trout included main and interactive effects of source-stream temperature and experimental temperature, but no effects of body mass for both (a) *Radix balthica* (Linear regression: $y = 0.0490 + 0.0013 \times T\text{-}T_0/kTT_0 + 0.04552 \times S_{tepid} + 0.0048 \times S_{warm} + 0.0664 \times T\text{-}T_0/kTT_0 \times S_{tepid} + 0.0419 \times T\text{-}T_0/kTT_0 \times S_{warm}$; $F_{5,73} = 4.222$; $p = 0.002$; $r^2 = 0.17$) and (b) *Simulium vittatum* (Linear regression: $y = 0.5011 - 0.0164 \times T\text{-}T_0/kTT_0 - 0.0512 \times S_{tepid} - 0.2106 \times S_{warm} + 0.1097 \times T\text{-}T_0/kTT_0 \times S_{tepid} + 0.1232 \times T\text{-}T_0/kTT_0 \times S_{warm}$; $F_{5,82} = 9.609$; $p < 0.001$; $r^2 = 0.33$). The graphical legend indicates the stream of origin for each population: blue = IS12, orange = IS1, and red = IS5. Note that for *Radix balthica*, the slope of IS12 (0.0013 ± 0.0317; mean ± 95% CI) was significantly different from IS1 (0.0677 ± 0.0557; $t = 2.39$, $p = 0.020$), but not IS5 (0.0432 ± 0.0544; $t = 1.54$, $p = 0.127$), while IS1 was not significantly different from IS5 ($t = -0.77$, $p = 0.443$). For *Simulium vittatum*, the slope of IS12 (-0.0164 ± 0.0602; mean ± 95% CI) was significantly different from both IS1 (0.0932 ± 0.1032; $t = 2.13$, $p = 0.037$) and IS5 (0.1068 ± 0.1056; $t = 2.33$, $p = 0.022$), while IS1 was not significantly different from IS5 ($t = 0.23$, $p = 0.823$). Shaded areas are the 95% confidence intervals around the fitted regression lines.

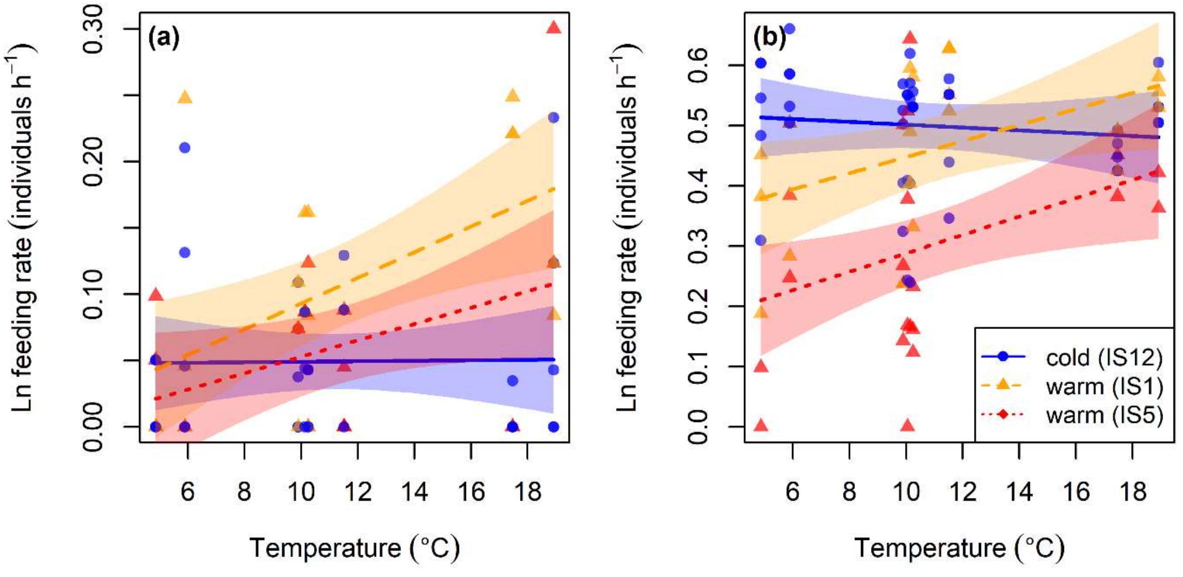

Supplementary Figure 3. Effects of source-stream temperature on energetic efficiency considering three separate populations of trout. The optimum model describing variation in the energetic efficiency of brown trout included a main effect of body mass and main and interactive effects of source-stream temperature and experimental temperature for both (a) *Radix balthica* (Linear regression: $y = 3.972 - 0.7533 \times \log(M) - 0.3592 \times T\text{-}T_0/kTT_0 + 0.4075 \times S_{tepid} - 0.0325 \times S_{warm} + 0.8985 \times T\text{-}T_0/kTT_0 \times S_{tepid} + 0.4442 \times T\text{-}T_0/kTT_0 \times S_{warm}$; $F_{6,79} = 23.9$; $p < 0.001$; $r^2 = 0.62$) and (b) *Simulium vittatum* (Linear regression: $y = 7.17 - 0.7494 \times \log(M) - 0.3825 \times T\text{-}T_0/kTT_0 - 0.3067 \times S_{tepid} - 0.9192 \times S_{warm} + 0.4016 \times T\text{-}T_0/kTT_0 \times S_{tepid} + 0.3901 \times T\text{-}T_0/kTT_0 \times S_{warm}$; $F_{6,79} = 40.42$; $p < 0.001$; $r^2 = 0.74$). The graphical legend indicates the stream of origin for each population: blue = IS12, orange = IS1, and red = IS5. Note that for *Radix balthica*, the slope of IS12 (-0.3592 ± 0.0999; mean ± 95% CI) was significantly different from both IS1 (0.5395 ± 0.3228; $t = 5.57$, $p < 0.001$) and IS5 (0.0850 ± 0.3833; $t = 2.32$, $p = 0.023$), while IS1 was not significantly different from IS5 ($t = -1.46$, $p = 0.148$). For *Simulium vittatum*, the slope of IS12 (-0.3825 ± 0.1004; mean ± 95% CI) was significantly different from both IS1 (0.0191 ± 0.3246; $t = 2.47$, $p = 0.016$) and IS5 (0.0076 ± 0.3855; $t = 2.02$, $p = 0.046$), while IS1 was not significantly different from IS5 ($t = -0.055$, $p = 0.956$). Shaded areas are the 95% confidence intervals around the fitted regression lines.

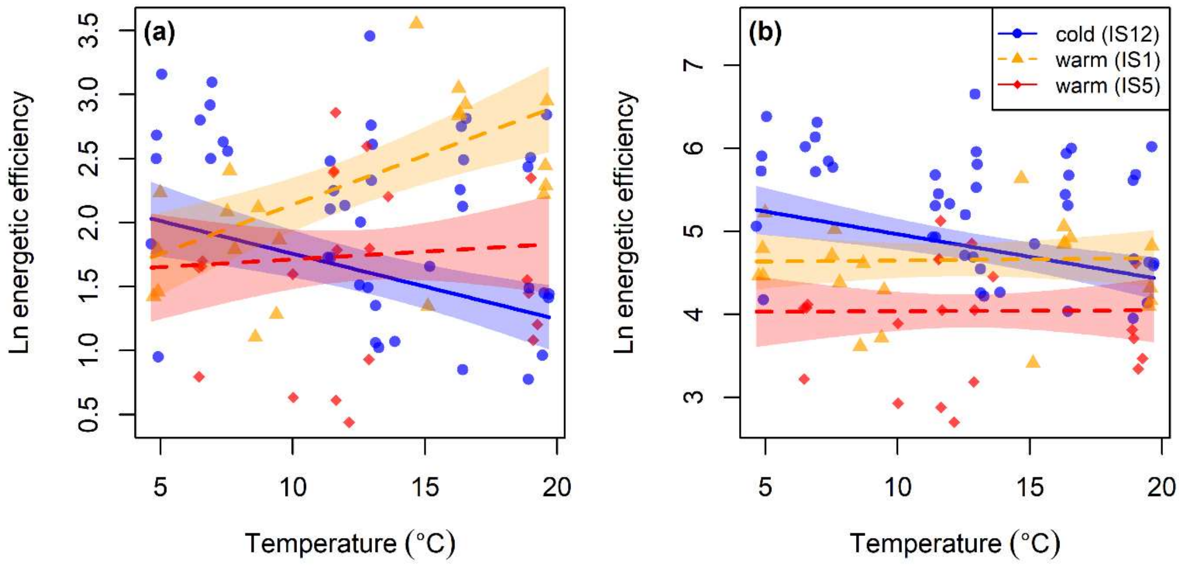

Supplementary Figure 4. Genetic clustering of fish from the study streams. PCA-based *k*-means clustering identified the optimum number of clusters in the genetics data as two, which are visualised here using discriminant function analysis of principle components (DAPC). The inset pie charts show the proportion of fish from the three study streams assigned to each cluster: blue = IS12, orange = IS1, and red = IS5.

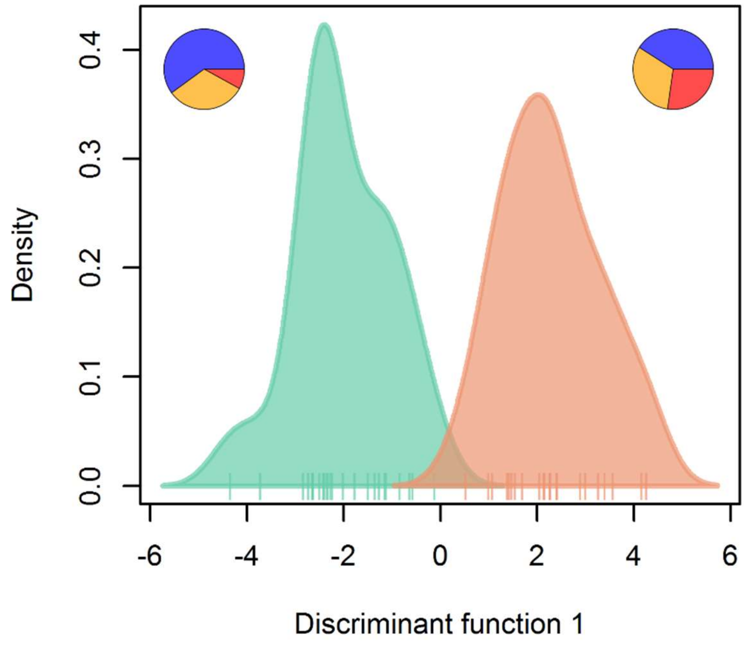

Supplementary Figure 5. Similarity in key environmental variables apart from temperature in the three study streams. 12 environmental variables were sampled in the three study streams in August 2004, August 2008, and April 2009 (see references 87 and 89 in the main text for methodological details). There was no significant difference in the concentration of pH ($F_{1,7}$ = 0.125, p = 0.733), total nitrogen ($F_{1,7}$ = 0.854, p = 0.386), total phosphorous ($F_{1,7}$ = 0.960, p = 0.360), ammonia ($F_{1,7}$ = 0.933, p = 0.366), nitrate ($F_{1,7}$ = 0.045, p = 0.838), phosphate ($F_{1,7}$ = 0.255, p = 0.629), sulphate ($F_{1,7}$ = 0.800, p = 0.401), calcium ($F_{1,7}$ = 0.425, p = 0.535), potassium ($F_{1,7}$ = 1.046, p = 0.340), magnesium ($F_{1,7}$ = 0.008, p = 0.933), sodium ($F_{1,7}$ = 1.941, p = 0.206), or chlorine ($F_{1,7}$ = 0.135, p = 0.724) among the three streams. Tukey method box and whisker plots are displayed, where the thick black lines are the median values for each class, the outer margins of the box are the first and third quartiles, and the whiskers are 1.5 times the interquartile range above or below the margins.

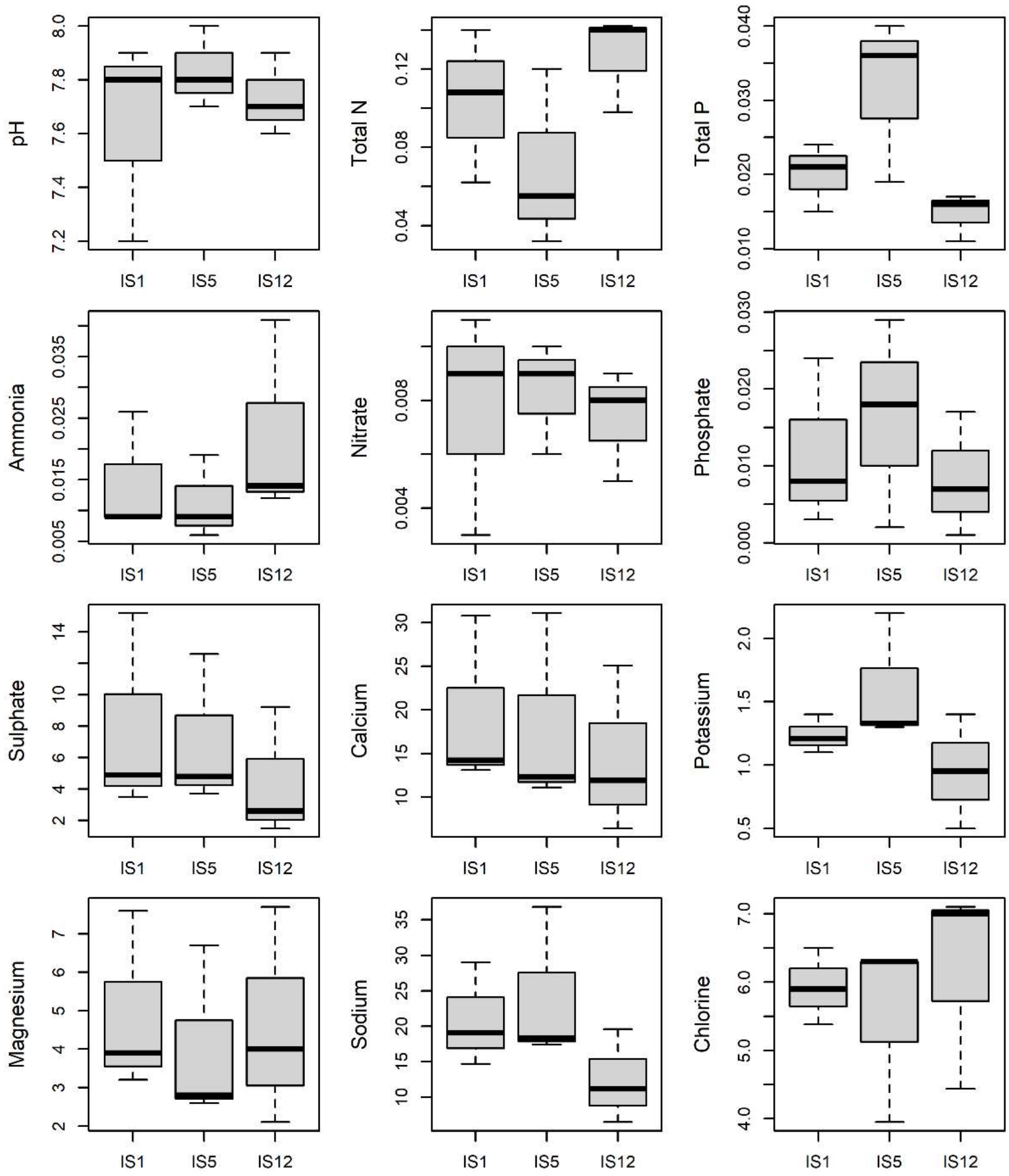
pH
Total N
Total P
Ammonia
Nitrate
Phosphate
Sulphate
Calcium
Potassium
Magnesium
Sodium
Chlorine
IS1
IS5
IS12

Supplementary Figure 6a. Dissolved oxygen concentrations during the experimental period. The first 30 minutes of each experiment (*i.e.* the acclimation period) are not visualised. Grey points and regression lines indicate experiments which were excluded due to $r^2 < 0.8$. Every individual panel is a different fish.

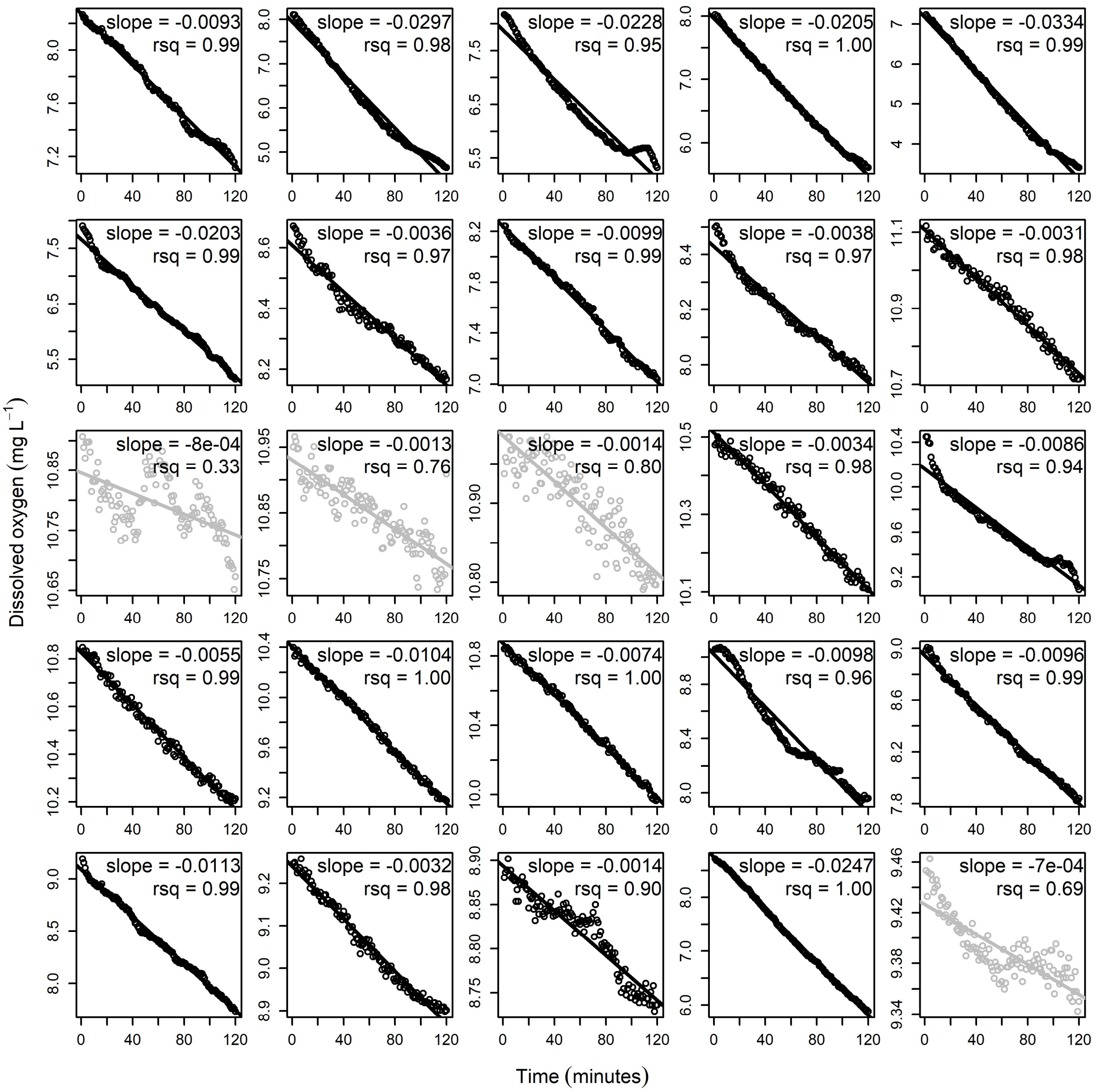

Supplementary Figure 6b. Dissolved oxygen concentrations during the experimental period. The first 30 minutes of each experiment (*i.e.* the acclimation period) are not visualised. Every individual panel is a different fish.

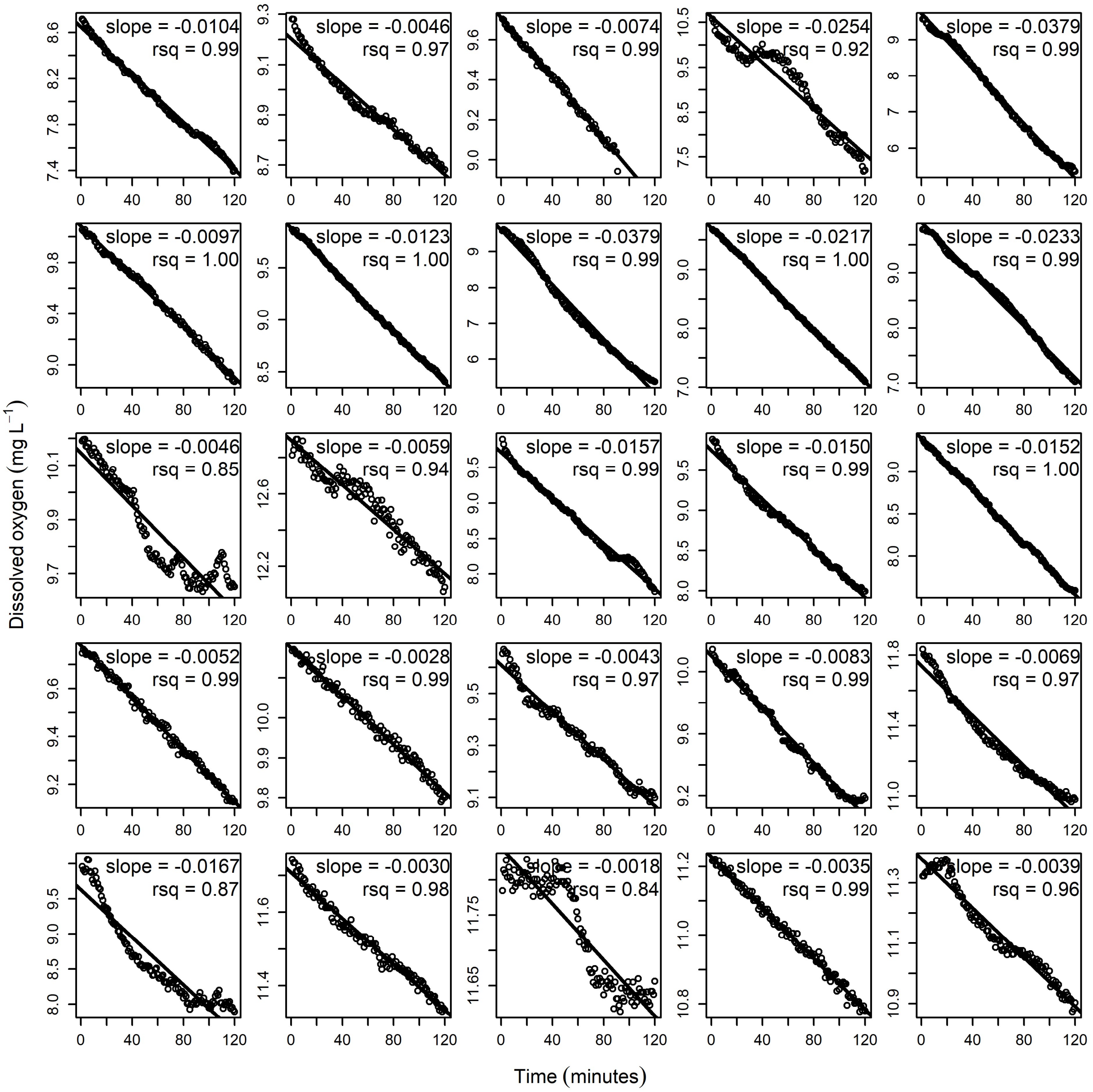

Supplementary Figure 6c. Dissolved oxygen concentrations during the experimental period. The first 30 minutes of each experiment (*i.e.* the acclimation period) are not visualised. Grey points and regression lines indicate experiments which were excluded due to $r^2 < 0.8$. Every individual panel is a different fish.

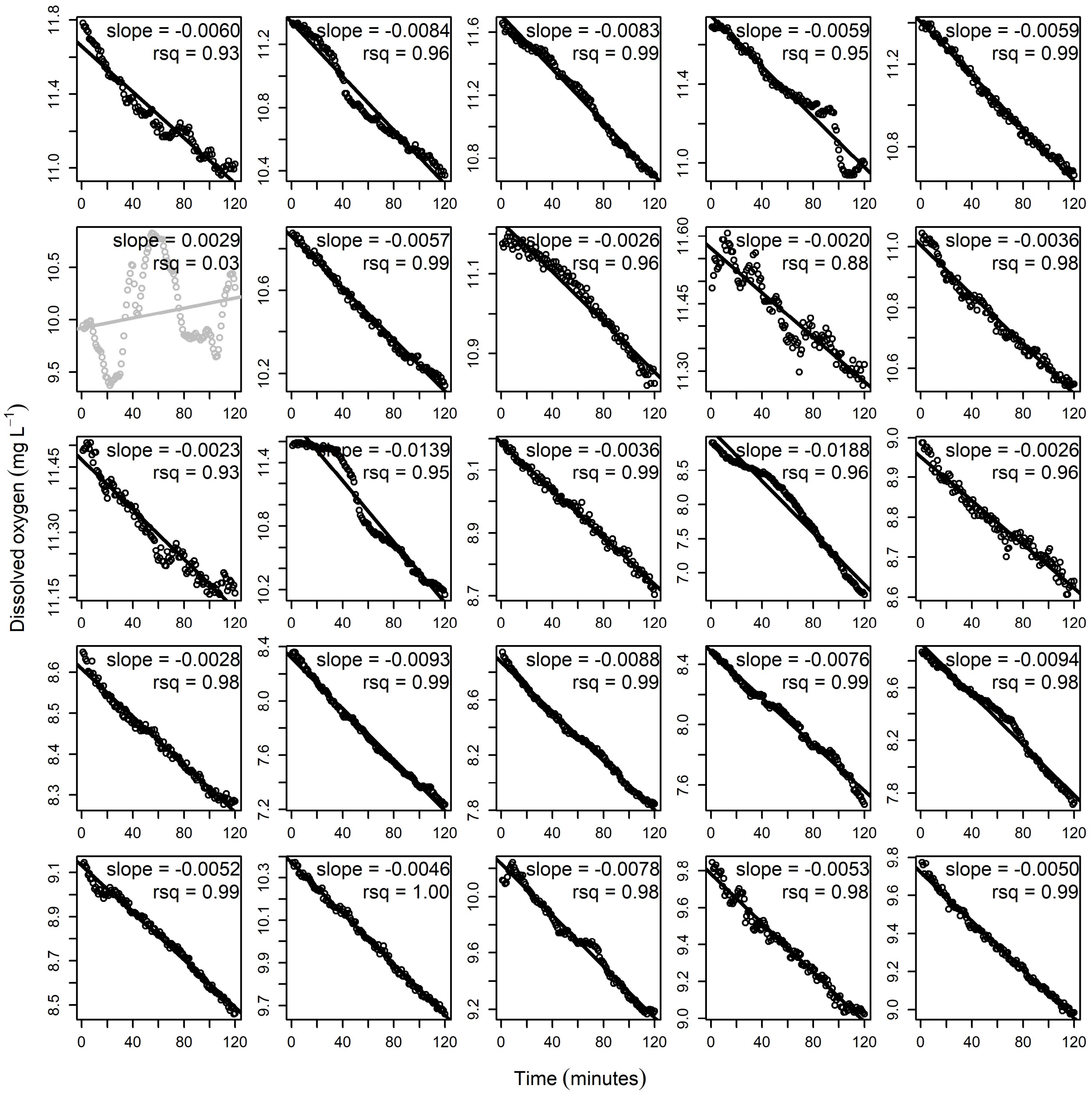

Supplementary Figure 6d. Dissolved oxygen concentrations during the experimental period. The first 30 minutes of each experiment (*i.e.* the acclimation period) are not visualised. Every individual panel is a different fish.

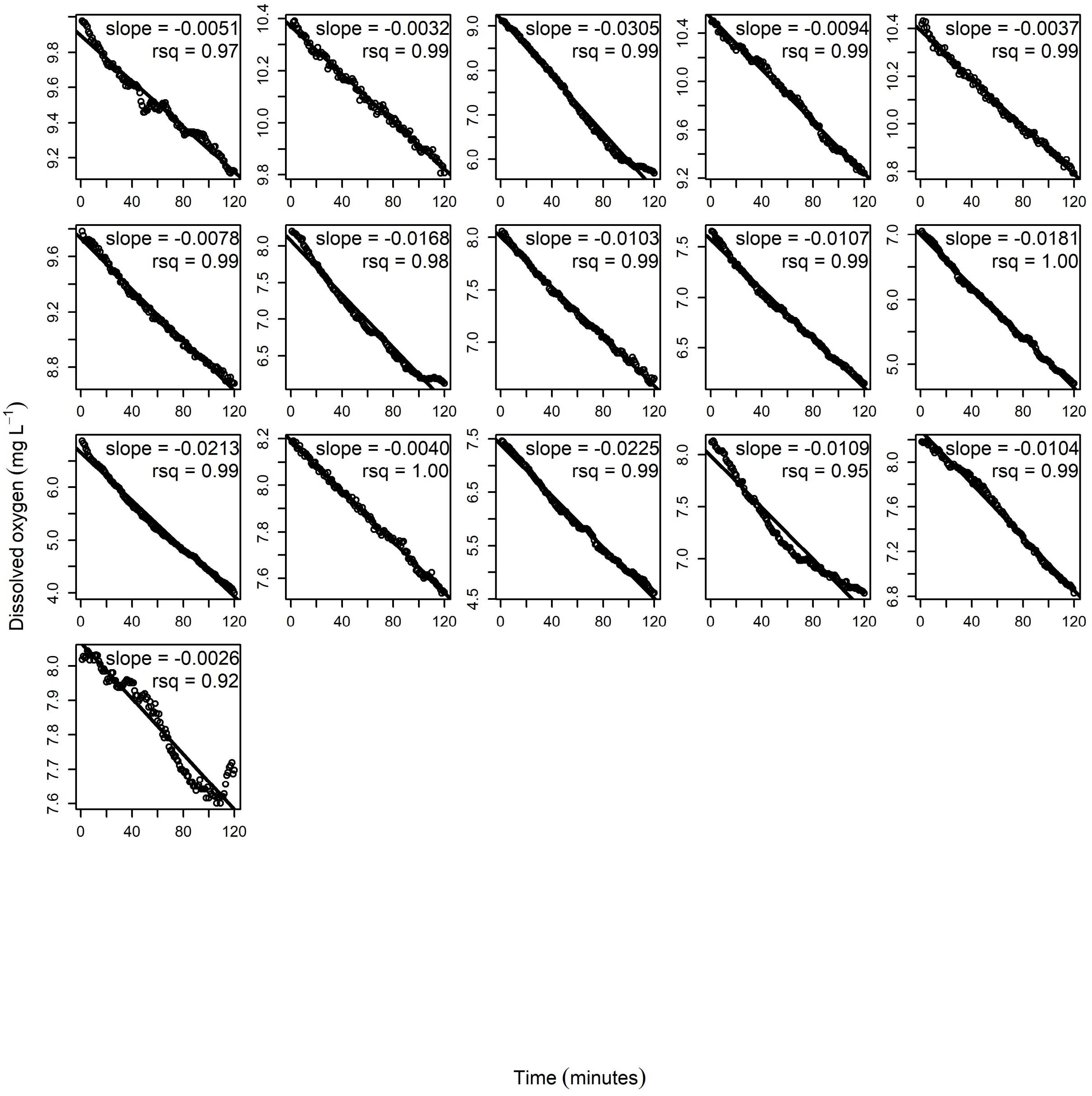

Supplementary Table 1. Comparison of models for determining effects of body mass, temperature, and source-stream temperature on metabolic rate. The identity (ID) and structure of each model are provided, with body mass (*M*), temperature (*T*), and source-stream temperature (*S*) following the form defined in Equation 1. The degrees of freedom (df), Akaike Information Criterion corrected for small sample size (AICc), and difference in AICc relative to the most parsimonious model (ΔAICc) are also displayed. Note that there was similar support for two other models (ΔAIC < 2), but the interaction term in *m8* and the source-stream temperature term in *m12* were not significant ($p = 0.211$ and $p = 0.232$, respectively), so we present the results of *m13*.

Model ID	Fixed effects	df	AICc	ΔAICc
m1	ln(M) + T + S + ln(M):T + ln(M):S + T:S + ln(M):T:S	9	119.8	8.1
m2	ln(M) + T + S + ln(M):T + ln(M):S + T:S	8	117.9	6.2
m3	ln(M) + T + S + ln(M):T + ln(M):S	7	115.5	3.7
m4	ln(M) + T + S + ln(M):T + T:S	7	117.1	5.4
m5	ln(M) + T + S + ln(M):S + T:S	7	115.5	3.8
m6	ln(M) + T + S + ln(M):T	6	114.7	3.0
m7	ln(M) + T + ln(M):T	5	114.0	2.3
m8	ln(M) + T + S + ln(M):S	6	113.1	1.4
m9	ln(M) + S + ln(M):S	5	136.2	24.5
m10	ln(M) + T + S + T:S	6	114.8	3.0
m11	T + S + T:S	5	177.6	65.9
m12	ln(M) + T + S	5	112.5	0.7
m13	ln(M) + T	4	111.7	0
m14	ln(M) + S	4	135.7	23.9
m15	T + S	4	175.4	63.7
m16	ln(*M*)	3	134.0	22.3
m17	T	3	197.2	85.5
m18	S	3	187.2	75.5
m19	none	2	204.2	92.5

Supplementary Table 2. Statistical output of the metabolic rate model considering three separate populations of trout. The optimum model describing variation in the metabolic rate of brown trout included main effects of body mass (*M*) and temperature (*T*), but no effect of source-stream temperature (*S*), following the form defined in Equation 1.

**Parameter**	**DF**	**SS**	***MS***	***F* value**	***p* value**
M	1	29.333	29.333	145.61	<0.001
T	1	5.5152	5.5152	27.377	<0.001
Residuals	83	16.721	0.2015		

Supplementary Table 3. Comparison of models for determining effects of body mass, temperature, and source-stream temperature on feeding rate of brown trout on *Radix balthica*. The identity (ID) and structure of each model are provided, with body mass (*M*), temperature (*T*), and source-stream temperature (*S*) following the form defined in Equation 1. The degrees of freedom (df), Akaike Information Criterion corrected for small sample size (AICc), and difference in AICc relative to the most parsimonious model (ΔAICc) are also displayed.

Model ID	Fixed effects	df	AICc	ΔAICc
m1	ln(M) + T + S + ln(M):T + ln(M):S + T:S + ln(M):T:S	9	-186.5	8.6
m2	ln(M) + T + S + ln(M):T + ln(M):S + T:S	8	-188.2	6.8
m3	ln(M) + T + S + ln(M):T + ln(M):S	7	-186.6	8.4
m4	ln(M) + T + S + ln(M):T + T:S	7	-190.5	4.5
m5	ln(M) + T + S + ln(M):S + T:S	7	-190.6	4.5
m6	ln(M) + T + S + ln(M):T	6	-188.8	6.2
m7	ln(M) + T + ln(M):T	5	-189.5	5.6
m8	ln(M) + T + S + ln(M):S	6	-187.5	7.6
m9	ln(M) + S + ln(M):S	5	-184.4	10.6
m10	ln(M) + T + S + T:S	6	-192.8	2.3
m11	T + S + T:S	5	-195.0	0
m12	ln(M) + T + S	5	-189.8	5.3
m13	ln(M) + T	4	-189.8	5.2
m14	ln(M) + S	4	-186.3	8.7
m15	T + S	4	-191.9	3.1
m16	ln(*M*)	3	-185.5	9.6
m17	T	3	-190.0	5.0
m18	S	3	-188.5	6.5
m19	none	2	-186.8	8.2

Supplementary Table 4. Comparison of models for determining effects of body mass, temperature, and source-stream temperature on feeding rate of brown trout on *Simulium vittatum*. The identity (ID) and structure of each model are provided, with body mass (*M*), temperature (*T*), and source-stream temperature (*S*) following the form defined in Equation 1. The degrees of freedom (df), Akaike Information Criterion corrected for small sample size (AICc), and difference in AICc relative to the most parsimonious model (ΔAICc) are also displayed.

Model ID	Fixed effects	df	AICc	ΔAICc
m1	ln(M) + T + S + ln(M):T + ln(M):S + T:S + ln(M):T:S	9	-86.0	7.8
m2	ln(M) + T + S + ln(M):T + ln(M):S + T:S	8	-87.4	6.3
m3	ln(M) + T + S + ln(M):T + ln(M):S	7	-86.3	7.4
m4	ln(M) + T + S + ln(M):T + T:S	7	-89.8	3.9
m5	ln(M) + T + S + ln(M):S + T:S	7	-89.2	4.5
m6	ln(M) + T + S + ln(M):T	6	-88.6	5.1
m7	ln(M) + T + ln(M):T	5	-76.1	17.6
m8	ln(M) + T + S + ln(M):S	6	-85.0	8.7
m9	ln(M) + S + ln(M):S	5	-83.8	9.9
m10	ln(M) + T + S + T:S	6	-91.5	2.2
m11	T + S + T:S	5	-93.7	0
m12	ln(M) + T + S	5	-87.3	6.4
m13	ln(M) + T	4	-76.4	17.4
m14	ln(M) + S	4	-86.0	7.7
m15	T + S	4	-89.1	4.7
m16	ln(*M*)	3	-75.7	18.0
m17	T	3	-77.3	16.4
m18	S	3	-87.8	5.9
m19	none	2	-76.5	17.2

Supplementary Table 5. Statistical output of the feeding rate model considering three separate populations of trout. The optimum models describing variation in the feeding rate of brown trout on both *Radix balthica* and *Simulium vittatum* included main effects of temperature (*T*), source-stream temperature (*S*), and their interaction (*T*:*S*), but no effect of body mass (*M*), following the form defined in Equation 1.

**Species**	**Parameter**	**DF**	**SS**	***MS***	***F* value**	***p* value**
Radix balthica	*T*	1	0.0269	0.0269	6.133	0.0156
	S	2	0.0379	0.0190	4.327	0.0168
	T:S	2	0.0277	0.0139	3.161	0.0482
	Residuals	73	0.3198	0.0044		
Simulium vittatum	*T*	1	0.0683	0.0683	4.308	0.0411
	S	2	0.5742	0.2871	18.12	<0.001
	T:S	2	0.1187	0.0594	3.747	0.0277
	Residuals	82	1.2991	0.0158		

Supplementary Table 6. Comparison of models for determining effects of body mass, temperature, and source-stream temperature on energetic efficiency of brown trout feeding on *Radix balthica*. The identity (ID) and structure of each model are provided, with body mass (*M*), temperature (*T*), and source-stream temperature (*S*) following the form defined in Equation 1. The degrees of freedom (df), Akaike Information Criterion corrected for small sample size (AICc), and difference in AICc relative to the most parsimonious model (ΔAICc) are also displayed. Note that there was similar support for *m5* (ΔAIC < 2), but the interaction term between body mass and source-stream temperature was not significant (p = 0.247), so we present the results of *m10*.

Model ID	Fixed effects	df	AICc	ΔAICc
m1	ln(M) + T + S + ln(M):T + ln(M):S + T:S + ln(M):T:S	9	119.6	5.0
m2	ln(M) + T + S + ln(M):T + ln(M):S + T:S	8	117.4	2.8
m3	ln(M) + T + S + ln(M):T + ln(M):S	7	132.8	18.2
m4	ln(M) + T + S + ln(M):T + T:S	7	116.9	2.3
m5	ln(M) + T + S + ln(M):S + T:S	7	115.1	0.5
m6	ln(M) + T + S + ln(M):T	6	131.6	17.0
m7	ln(M) + T + ln(M):T	5	141.5	26.9
m8	ln(M) + T + S + ln(M):S	6	136.7	22.1
m9	ln(M) + S + ln(M):S	5	134.3	19.7
m10	ln(M) + T + S + T:S	6	114.6	0
m11	T + S + T:S	5	178.1	63.5
m12	ln(M) + T + S	5	135.4	20.8
m13	ln(M) + T	4	143.2	28.6
m14	ln(M) + S	4	133.1	18.5
m15	T + S	4	186.2	71.6
m16	ln(*M*)	3	141.1	26.5
m17	T	3	185.3	70.7
m18	S	3	184.1	69.5
m19	none	2	183.2	68.6

Supplementary Table 7. Comparison of models for determining effects of body mass, temperature, and source-stream temperature on energetic efficiency of brown trout feeding on *Simulium vittatum*. The identity (ID) and structure of each model are provided, with body mass (*M*), temperature (*T*), and source-stream temperature (*S*) following the form defined in Equation 1. The degrees of freedom (df), Akaike Information Criterion corrected for small sample size (AICc), and difference in AICc relative to the most parsimonious model (ΔAICc) are also displayed. Note that there was similar support for *m5* (ΔAIC < 2), but the interaction term between body mass and source-stream temperature was not significant (p = 0.303), so we present the results of *m10*.

Model ID	Fixed effects	df	AICc	ΔAICc
m1	ln(M) + T + S + ln(M):T + ln(M):S + T:S + ln(M):T:S	9	119.7	5.0
m2	ln(M) + T + S + ln(M):T + ln(M):S + T:S	8	117.8	3.1
m3	ln(M) + T + S + ln(M):T + ln(M):S	7	122.6	7.9
m4	ln(M) + T + S + ln(M):T + T:S	7	117.0	2.4
m5	ln(M) + T + S + ln(M):S + T:S	7	115.3	0.7
m6	ln(M) + T + S + ln(M):T	6	121.5	6.8
m7	ln(M) + T + ln(M):T	5	133.2	18.6
m8	ln(M) + T + S + ln(M):S	6	123.0	8.3
m9	ln(M) + S + ln(M):S	5	125.5	10.8
m10	ln(M) + T + S + T:S	6	114.7	0
m11	T + S + T:S	5	177.6	63.0
m12	ln(M) + T + S	5	121.9	7.2
m13	ln(M) + T	4	134.4	19.7
m14	ln(M) + S	4	124.6	9.9
m15	T + S	4	179.2	64.6
m16	ln(*M*)	3	135.5	20.8
m17	T	3	220.2	105.6
m18	S	3	180.1	65.4
m19	none	2	219.1	104.4

Supplementary Table 8. Statistical output of the energetic efficiency model considering three separate populations of trout. The optimum models describing variation in the energetic efficiency of brown trout feeding on both *Radix balthica* and *Simulium vittatum* included main effects of body mass (*M*), temperature (*T*), source-stream temperature (*S*), and their interaction (*T*:*S*), following the form defined in Equation 1.

Species	Parameter	DF	SS	*MS*	*F* value	*p* value
Radix balthica	*M*	1	16.617	16.617	82.62	<0.001
	T	1	0.0262	0.0262	0.131	0.7189
	S	2	5.9071	2.9535	14.68	<0.001
	T:S	2	6.2944	3.1472	15.65	<0.001
	Residuals	79	15.890	0.2011		
Simulium vittatum	*M*	1	39.290	39.290	193.1	<0.001
	T	1	1.2890	1.2890	6.338	0.0138
	S	2	7.1730	3.5870	17.63	<0.001
	T:S	2	1.5860	0.7930	3.898	0.0243
	Residuals	79	16.071	0.2030		

Supplementary Table 9. Summary statistics on within-stream genetic diversity. HWE = Hardy Weinberg Equilibrium. Final column gives overall statistics across all 17 microsatellite loci combined.

Stream	Statistic	*Ssa416*	*One103*	*CocI-Lav-4*	*One9uASC*	*CA048828*	*One108*	*Ssa85*	*One102-a*	*One102-b*	*CA053293*	*ppStr2*	*ppStr3*	*CA060177*	*Ssa197*	*SsaD71*	*SaSaTAP2A*	*Ssa410UOS*	Overall
IS12	Number of samples	24	24	24	24	24	24	24	24	24	24	24	24	24	24	24	24	24	24
	Number of alleles	3	4	4	3	7	6	3	2	7	3	5	2	3	1	3	2	5	63
	Allelic richness	2.76	3.28	3.47	2.78	5.68	5.49	2.99	1.88	4.45	3.00	4.20	1.75	2.97	1.00	2.94	1.48	4.59	3.22
	Observed heterozygosity	0.75	0.54	0.62	0.58	0.67	0.75	0.67	0.12	0.79	0.83	0.58	0.17	0.58	0.00	0.62	0.08	0.75	0.54
	Expected heterozygosity	0.57	0.64	0.55	0.52	0.79	0.82	0.65	0.30	0.69	0.66	0.65	0.15	0.63	0.00	0.62	0.08	0.74	0.53
	HWE exact test *p*-value	0.54	0.05	0.59	0.93	0.26	1.00	1.00	0.01	0.29	0.03	0.52	1.00	0.92	NA	0.72	1.00	1.00	0.55
IS1	Number of samples	15	15	15	15	15	15	15	15	15	15	15	15	15	15	15	15	15	15
	Number of alleles	2	4	4	3	6	7	4	2	6	3	4	2	3	2	3	2	5	62
	Allelic richness	2.00	3.60	3.63	2.76	5.42	5.76	3.10	1.85	5.16	2.96	3.86	1.83	3.00	1.40	2.41	1.83	4.29	3.23
	Observed heterozygosity	0.53	0.67	0.53	0.47	0.67	0.67	0.53	0.20	0.87	0.80	0.60	0.13	0.80	0.07	0.60	0.20	0.80	0.54
	Expected heterozygosity	0.50	0.65	0.55	0.38	0.80	0.81	0.59	0.18	0.79	0.58	0.72	0.23	0.66	0.06	0.46	0.18	0.73	0.52
	HWE exact test *p*-value	1.00	1.00	0.71	0.55	0.09	0.96	0.44	1.00	0.76	0.33	0.81	0.25	0.78	1.00	0.05	1.00	0.78	0.95
IS5	Number of samples	8	8	8	8	8	8	8	8	8	8	8	8	8	8	8	8	8	8
	Number of alleles	2	4	4	3	4	7	3	2	5	3	4	2	3	1	3	2	5	57
	Allelic richness	2.00	3.62	3.31	2.57	3.82	6.09	2.69	1.65	4.27	2.98	3.90	1.66	2.98	1.00	2.65	1.67	4.64	3.03
	Observed heterozygosity	0.50	0.62	0.50	0.25	0.62	1.00	0.62	0.12	0.62	0.62	1.00	0.12	0.62	0.00	0.62	0.12	1.00	0.53
	Expected heterozygosity	0.47	0.65	0.60	0.32	0.66	0.83	0.55	0.12	0.72	0.62	0.72	0.12	0.63	0.00	0.46	0.12	0.77	0.49
	HWE exact test *p*-value	1.00	1.00	0.48	0.14	0.95	0.07	0.48	1.00	0.05	0.01	0.74	1.00	1.00	NA	1.00	1.00	1.00	0.73
