# Supplementary material for: Brown trout (Salmo trutta) originating from warmer streams in Iceland exhibit increased energetic efficiency
Source: Commun Biol. 2026 Mar 31;9:710. doi: 10.1038/s42003-026-09911-5 (PMC13201538; doi:10.1038/s42003-026-09911-5)
Supplement: Supplementary file 3 — Description of Additional Supplementary files [file 42003_2026_9911_MOESM3_ESM.pdf]

Description of Additional Supplementary files

File name: Supplementary Data

Description: Supplementary Data for the the figures in the O'Gorman et al article "Brown trout (Salmo trutta) originating from warmer streams in Iceland exhibit increased energetic efficiency"
