# Supplementary material for: Brown trout (Salmo trutta) originating from warmer streams in Iceland exhibit increased energetic efficiency
Source: Commun Biol. 2026 Mar 31;9:710. doi: 10.1038/s42003-026-09911-5 (PMC13201538; doi:10.1038/s42003-026-09911-5)
Supplement: Supplementary file 5 — Reporting Summary [file 42003_2026_9911_MOESM5_ESM.pdf]

nature portfolio

Corresponding author(s): Eoin J. O'Gorman

Last updated by author(s): Dec 4, 2024

Reporting Summary

Nature Portfolio wishes to improve the reproducibility of the work that we publish. This form provides structure for consistency and transparency in reporting. For further information on Nature Portfolio policies, see our Editorial Policies and the Editorial Policy Checklist.

Statistics

For all statistical analyses, confirm that the following items are present in the figure legend, table legend, main text, or Methods section.

n/a	Confirmed	
☐	☒	The exact sample size (*n*) for each experimental group/condition, given as a discrete number and unit of measurement
☐	☒	A statement on whether measurements were taken from distinct samples or whether the same sample was measured repeatedly
☐	☒	The statistical test(s) used AND whether they are one- or two-sided<br>*Only common tests should be described solely by name; describe more complex techniques in the Methods section.*
☐	☒	A description of all covariates tested
☐	☒	A description of any assumptions or corrections, such as tests of normality and adjustment for multiple comparisons
☐	☒	A full description of the statistical parameters including central tendency (e.g. means) or other basic estimates (e.g. regression coefficient) AND variation (e.g. standard deviation) or associated estimates of uncertainty (e.g. confidence intervals)
☐	☒	For null hypothesis testing, the test statistic (e.g. *F*, *t*, *r*) with confidence intervals, effect sizes, degrees of freedom and *P* value noted<br>*Give P values as exact values whenever suitable.*
☒	☐	For Bayesian analysis, information on the choice of priors and Markov chain Monte Carlo settings
☒	☐	For hierarchical and complex designs, identification of the appropriate level for tests and full reporting of outcomes
☒	☐	Estimates of effect sizes (e.g. Cohen's *d*, Pearson's *r*), indicating how they were calculated

Our web collection on statistics for biologists contains articles on many of the points above.

Software and code

Policy information about availability of computer code

Data collection	No software was used to collect the data.
Data analysis	All statistical analyses were carried out in R 4.0.2.

For manuscripts utilizing custom algorithms or software that are central to the research but not yet described in published literature, software must be made available to editors and reviewers. We strongly encourage code deposition in a community repository (e.g. GitHub). See the Nature Portfolio guidelines for submitting code & software for further information.

Data

Policy information about availability of data

All manuscripts must include a data availability statement. This statement should provide the following information, where applicable:

- Accession codes, unique identifiers, or web links for publicly available datasets
- A description of any restrictions on data availability
- For clinical datasets or third party data, please ensure that the statement adheres to our policy

The data that support the findings of this study will be uploaded to the University of Essex Data Repository upon acceptance of the article.

Research involving human participants, their data, or biological material

Policy information about studies with human participants or human data. See also policy information about sex, gender (identity/presentation), and sexual orientation and race, ethnicity and racism.

Reporting on sex and gender	N/a
Reporting on race, ethnicity, or other socially relevant groupings	N/a
Population characteristics	N/a
Recruitment	N/a
Ethics oversight	N/a

Note that full information on the approval of the study protocol must also be provided in the manuscript.

Field-specific reporting

Please select the one below that is the best fit for your research. If you are not sure, read the appropriate sections before making your selection.

☐ Life sciences ☐ Behavioural & social sciences ☒ Ecological, evolutionary & environmental sciences

For a reference copy of the document with all sections, see nature.com/documents/nr-reporting-summary-flat.pdf

Ecological, evolutionary & environmental sciences study design

All studies must disclose on these points even when the disclosure is negative.

Study description	Metabolic rate and feeding rate experiments were carried out in five geothermally heated streams in the Hengill valley, Iceland. Brown trout (Salmo trutta) were collected from a cold stream and two warm streams and then translocated to streams of different temperature in the system for the subsequent experiments. Oxygen consumption rates were used as a proxy for metabolic rate using a MiniDOT logger (PME, USA) placed inside 7.2 L circular plastic chambers (LocknLock, South Korea) with lids that could be opened and closed to create an airtight seal. Before placement in the streams, each chamber was fully submerged in a 50 L plastic container that had been filled with water from the experimental stream, filtered through a 250 µm sieve to remove small organisms or plant matter that may affect the level of background respiration. A single brown trout individual was placed inside each chamber along with the MiniDOT logger. The chamber was then sealed underwater to avoid any air bubbles that may interfere with dissolved oxygen readings and completely submerged in the experimental stream, with a rock on the lid to secure it to the stream bed. Up to ten chambers containing fish were placed in the stream for each experimental run, whilst an extra chamber without any fish was included as a control for measuring background respiration. The experiments ran for at least 2.5 hours, after which time the fish was removed, its body length was measured, and it was subsequently used in a feeding rate experiment. Before each experiment, 200 freshwater snails (Radix balthica) and 200 blackfly larvae (Simulium vittatum) were hand-collected from an independent stream. Twenty individuals of a particular taxon were added to each of twenty cylindrical arenas (identical to those used to store fish before the metabolic experiments), with ten arenas containing snails and ten containing blackfly larvae. The arenas were secured in the experimental stream with metal rebars and rocks. An individual trout was then added to each arena, ensuring that five arenas for each prey taxon contained trout from the cold stream and five contained trout from the warm streams. The experiments ran for approximately 24 hours, after which time the remaining prey individuals were counted, and fish were released back into their home streams. Adipose fins were removed for genetic analysis prior to release and thus also ensuring that no fish would be reused in any further experiments.
Research sample	A total of 91 brown trout (65–180 mm fork length) were collected from three streams in the Hengill geothermal valley, Iceland, where they are particularly abundant: 47 from a cold stream (IS12 with a mean annual temperature of 7.8 ± 4.2 standard deviations °C); and 44 from two warm streams (IS1 = 11.3 ± 4.0 °C and IS5 = 13.8 ± 1.6 °C). All trout less than 65 mm were deemed too small for the metabolic rate experiments and thus released, but we otherwise collected every fish we sampled from electrofishing for use in the experiments. This ensures that the research sample is representative of the typical size, age, and sex structure in each stream. Similarly, we collected the first 200 freshwater snails (Radix balthica) and blackfly larvae (Simulium vittatum) we could find in IS7 for use in the feeding rate experiments and thus these research samples are also representative of the typical size, age, and sex structure of each macroinvertebrate species in the stream.
Sampling strategy	No sample-size calculation was performed, but the number of experiments conducted for metabolic rate (n = 86), feeding rate on Radix balthica (n = 79), and feeding rate on Simulium vittatum (n = 88) were all more than sufficient to ensure a large enough sample size for linear regression analysis (recommended minimum n = 30). Model validation was conducted on all analyses and the assumptions of normality, homogeneity, and independence of residuals were met in all cases.
Data collection	The samples were processed by Eoin O'Gorman and members of his research group, with O'Gorman collating and screening all the datasets into Excel files for subsequent statistical analysis.
Timing and spatial scale	The experiments were conducted from 20th May to 3rd June 2018 in the Hengill geothermal valley, Iceland. After some pilot work on

Timing and spatial scale	the first few days, the metabolism experiments were conducted over a total of five days with two experimental runs of up to 10 fish conducted on each day. Fish were used in two 24-hour feeding trials immediately after each metabolism experiment, with one feeding trial conducted with the freshwater snail Radix balthica and another conducted with the blackfly larva Simulium vittatum. At the end of these experiments, a fin clip was taken from the fish to ensure it would not be reused in any experiment and it was released back into its natal stream.
Data exclusions	For the metabolic rate experiments, we excluded any data where the linear regression of dissolved oxygen data during the experimental period had an r2 value < 0.8, which occurred in 5 out of 91 experiments. Figure S6 in Supporting Information provides a detailed breakdown of which experiments were excluded due to these criteria. No data were excluded from the feeding rate experiments.
Reproducibility	The experiment was a huge undertaking in a remote Icelandic valley, so no attempt has been made to repeat it or check the reproducibility of the findings.
Randomization	We ensured that we always included a balanced number of fish collected from cold and warm streams, e.g. if there were 10 metabolic experiments conducted, 5 would be on fish collected from a cold stream and 5 would be on fish collected from a warm stream. The fish for each experimental run were chosen at random from the storage containers in their natal streams.
Blinding	Samples were processed according to unique codes that were only translated into treatment identifiers once all the data were collected.

Did the study involve field work? ☒ Yes ☐ No

Field work, collection and transport

Field conditions	The study was conducted in the Hengill valley, Iceland, which consists of numerous spring-fed streams that occur within 1.5 km of each other and have similar physical and chemical properties, yet vary in mean annual temperature from 5-20 °C due to indirect heating of groundwater through the bedrock. Air temperatures ranged between 5-15 °C during the experiments, with frequent rainfall.
Location	Fieldwork was performed in the Hengill geothermal valley, Iceland (N 64°03; W 21°18) at 350-420 metres above sea level.
Access & import/export	Access to the field site was obtained through collaboration with local researchers from the University of Iceland (Prof Gísli Már Gíslason) and the Marine and Freshwater Research Institute (Dr Jón S Ólafsson). No export permits were required for shipping samples of preserved trout adipose fin clips from Iceland to the UK, other than filling out customs declaration documentation through the shipping company Eimskip.
Disturbance	The study caused minimal disturbance to the field site. Researchers were careful to stick to established walking trails and to avoid excessive trampling of the vegetation or streams during sampling. The sampling protocols were minimally invasive, with hand picking of invertebrates off rocks and electrofishing to capture fish, which were returned to their natal streams at the end of the experiments. Great care was taken not to cross-contaminate streams in the system.

Reporting for specific materials, systems and methods

We require information from authors about some types of materials, experimental systems and methods used in many studies. Here, indicate whether each material, system or method listed is relevant to your study. If you are not sure if a list item applies to your research, read the appropriate section before selecting a response.

Materials & experimental systems

n/a	Involved in the study
☒	☐ Antibodies
☒	☐ Eukaryotic cell lines
☒	☐ Palaeontology and archaeology
☐	☒ Animals and other organisms
☒	☐ Clinical data
☒	☐ Dual use research of concern
☒	☐ Plants

Methods

n/a	Involved in the study
☒	☐ ChIP-seq
☒	☐ Flow cytometry
☒	☐ MRI-based neuroimaging

Animals and other research organisms

Policy information about studies involving animals; ARRIVE guidelines recommended for reporting animal research, and Sex and Gender in Research

Laboratory animals	The study did not involve laboratory animals.

Wild animals	All fish were released back to their natal streams at the end of the experiment. Freshwater macroinvertebrates were used as potential prey for the fish in the feeding rate experiments. The two most abundant macroinvertebrate species in the system were used (the snail, Radix balthica, and the blackfly larva, Simulium vittatum) and in relatively small numbers compared to their natural abundance.
Reporting on sex	Sex of the fish or invertebrates was not considered in the study design because it was not deemed relevant to addressing the hypotheses under investigation.
Field-collected samples	No live organisms were collected at the end of the experiment and so no laboratory housing was needed.
Ethics oversight	Electrofishing and handling of brown trout in the experiment was performed in collaboration with the Marine and Freshwater Research Institute under their ethical guidelines, permits, and regulations.

Note that full information on the approval of the study protocol must also be provided in the manuscript.
